# The Microbes in Milk and Water

**Published:** 1889-09

**Authors:** 


### THE MICROBES IN MILK AND WATER.

It has been calculated that in a cubic centimetre of milk (about twenty drops) there may be between 2,000,000 and 3,000,000 microbes, and possibly hundreds or thousands of different kinds ; and the next twenty drops of milk may contain as many more varieties. The microbes in a drop of water taken from the well may consist in a number of straight rods ; at the end of an hour the rods break in two, and in another hour another division takes place, the number doubling about every hour. Every minute that the water is exposed to the air adds to it hundreds of microbes, and yet water is pronounced good or bad, according to what the analyst sees through his microscope, or thinks he sees when the water finally reaches him in his laboratory.—*New York Phar. Journal.*

